# Phase I Clinical Trial Using Autologous Ex Vivo Expanded NK Cells and Cytotoxic T Lymphocytes for Cancer Treatment in Vietnam

**DOI:** 10.3390/ijms20133166

**Published:** 2019-06-28

**Authors:** Nguyen Thanh Liem, Nguyen Van Phong, Nguyen Trung Kien, Bui Viet Anh, Truong Linh Huyen, Chu Thi Thao, Nguyen Dac Tu, Doan Trung Hiep, Do Thi Hoai Thu, Hoang Thi My Nhung

**Affiliations:** 1 Vinmec Research Institute of Stem Cell and Gene Technology, 458 Minh Khai, Hanoi 100000, Vietnam; 2 Faculty of Biology, VNU University of Science, 334 Nguyen Trai, Hanoi 100000, Vietnam

**Keywords:** immune cell therapy, natural killer cells, cytotoxic T lymphocytes, peripheral blood mononuclear cells, cancer

## Abstract

(1) Background: Immune cell therapy recently attracted enormous attention among scientists as a cancer treatment, but, so far, it has been poorly studied and applied in Vietnam. The aim of this study was to assess the safety of autologous immune cell therapy for treating lung, liver, and colon cancers—three prevalent cancers in Vietnam. (2) Method: This was an open-label, single-group clinical trial that included 10 patients with confirmed diagnosis of colon, liver, or lung cancer, conducted between March 2016 and December 2017. (3) Results: After 20–21 days of culture, the average number of cytotoxic T lymphocytes (CTLs) increased 488.5-fold and the average cell viability was 96.3%. The average number of natural killer cells (NKs) increased 542.5-fold, with an average viability of 95%. Most patients exhibited improved quality of life, with the majority of patients presenting a score of 1 to 2 in the Eastern Cooperative Oncology Group (ECOG) performance status (ECOG/PS) scale, a decrease in symptoms on fatigue scales, and an increase in the mean survival time to 18.7 months at the end of the study. (4) Conclusion: This method of immune cell expansion met the requirements for clinical applications in cancer treatment and demonstrated the safety of this therapy for the cancer patients in Vietnam.

## 1. Introduction

Cancer is not only one of the leading causes of death in the world, but the social and economic implications of the disease, other than its mortality, is also a global concern. Therefore, identifying novel therapies for the prevention and treatment of cancer is the first-rate concern of scientists around the world. Immune cell therapy is one of the approaches which attracts the attention of many scientists in recent years [1,2]. In this therapy, the aim is not only to directly kill cancer cells, but also to boost immune cells in the patient’s body to fight cancer cells [3,4,5,6]. The immune cells in the body are stimulated and activated to trigger their capacity of recognizing and killing cancer cells. The principle of this therapy is isolating immune cells from the patient’s peripheral blood, culturing them to expand the number of one or more kinds of cells in a specific medium, and finally transfusing the whole of these expanded cells back into patient’s body [7,8]. This therapy may utilize multiple different kinds of immune cell, including natural killer cells (NKs), cytotoxic T lymphocytes (CTLs), cytokine-induced killer cells (CIKs), or dendritic cells (DCs). 

Clinical studies and trials have identified a significant role of NK cells in cancer development. When the activity of peripheral blood NK cells decreases, the risk of having cancer in adults conversely increases [9]. NK cells are lymphocytes which are expressed by CD56 and are negative for CD3. The CD56 expression level of NK cells is varied. Appropriate 90% NK cells from peripheral blood and spleen have low expression of CD56 and high expression of CD16 (CD56dimCD16+). These cells have strong cytotoxicity when they interact with target cells, for example, cancer cells. Conversely, almost all NK cells from lymph nodes have high expressions of CD56 and do not express CD16, and they play a role in immune regulatory functions through the secretion of some cytokines, like interferon γ response to the stimulation by IL-12, IL-15 and IL-18 [10,11]. NK cells have the ability to realize an abnormal expression level of the major histocompatibility complex (MHC) class I on the surface, which helps NK cells to distinguish between normal cells and abnormal cells in the body. NK cells have the capacity to recognize cancer cells based on the analysis of factors, which are non-self, missing-self, or stress-induced self [12,13]. The activity of NK cells depends on the balance between inhibitory signals and activating signals through several different receptors on the surface [14,15]. Resting NK cells can be primed by dendritic cells and, upon on activation, they might in turn induce dendritic cell maturation [16,17,18].

Many studies have pointed out that cytotoxic T cells (CTLs) have the ability to kill cancer cells which are NK cell-resistant. CTLs could also attack cancer cells through an MHC-independent mechanism, killing autologous cancer cells without affecting other cells in the body. T lymphocytes have the ability to recognize strange antigens expressed on the surface of cancer cells [19,20,21]. Similar to NK cells, when recognizing alien cells activated T lymphocytes might trigger the apoptosis processes in the target cells. On the surface of T cells, there are T-cell receptors (TCRs), which have the capacity of linking specifically with corresponding antigens. When cancer cells express alien antigens, this attracts T lymphocytes to home in and link to target cells through TCRs and trigger apoptosis processes. CTLs are used for the treatment of multiple kinds of cancer, including melanoma, hepatic cancer, lung cancer, and gastric cancer. [22,23,24,25]. The effectiveness and safety of therapies employing CTLs has been reported. Today, this therapy is, for instance, broadly applied throughout Japan and recognized as an “advanced medical technology” by the Japanese Ministry of Health, Labor, and Welfare [24].

The combination of two kinds of immune cells, including NK cells and CTLs, promises to be advantageous in the detection and killing of cancer cells with or without MHC-I expression. Many clinical studies have demonstrated the efficacy of the combination between these two kinds of immune cells in pre-clinical research models and clinical trials [5,23,25,26,27,28,29,30,31,32]. Expansion of immune cells by using BINKIT, developed by Dr. Terunuma Hiroshi (Biotherapy Institute of Japan, Tokyo, Japan), were proven to efficiently induce the number of selective cells and activate them to kill cancer cells [25,26,27,28,29,30,31,32].

Lung, liver, and colon cancer are the three prevalent cancers in Vietnam with the highest incidence and mortality [33]. Patients with these cancers are normally diagnosed at a late stage and, therefore, are left with no effective curative treatment for such advanced stages. Moreover, the treatment outcomes for these cancers are still considered to be discouraging, rendering them to be among the most difficult ones to treat [33,34]. The aim of this study was to assess the safety of autologous immune enhancement therapy for treating lung, liver, and colon cancers—the prevalent cancers in Vietnam.

## 2. Results

### 2.1. Patient Characteristics

A total of 10 patients with cancer, including four males and six females, were enrolled in this study. The median age for all study subjects was 60.5 years (ranging from 30 to 82 years old). The number of patients who smoke was three, while the figure for drinking was two. The types of cancer diagnosed in these patients were: Seven patients with lung cancer, two patients with liver cancer, and one patient with colon cancer. All patients were evaluated at disease stage IV. Within this patient cohort, tissue metastasis was diagnosed in eight patients, two patients were diagnosed to be non-metastatic. The mean of estimated survival time upon joining the present study was 7.8 months (Table 1).

### 2.2. Immune Cell Expansion Ability

Patients enrolled in this study received different numbers of immune cell sittings, typically ranged from 1 to 4 sittings, and in one exceptional case 9 sitting (Table 2). The total cell number from each expansion was used, resulting in cell number ranging from 6.61 × 10^8^ to 3.12 × 10^10^ cells/patient in this study (Table 2). Peripheral blood of cancer patients was collected and cultured on the same day. At the time of collection, the mean number of white blood cells was 4.8 ± 2 × 10^6^/mL, among them, lymphocytes accounted for 26.2 ± 11.4% and monocytes for 7.0 ± 1.6%. The percentage of lymphocytes in mononuclear cells was 77.5 ± 8.7%. The isolated mononuclear cells were divided into two equal parts for NK cell expansion (NK cell culture) and CTL expansion (CTL culture). At the seeding of the culture, the total number of cells ranged from 21.2 to 131.5 × 10^6^, NK cells accounted for 18% with the absolute number ranging from 1.9 to 35.7 × 10^6^, and CTLs accounted for 19% with the absolute number ranging from 2.3 to 21.8 × 10^6^.

At days 20–21 of culture, the number and cell component in each culture condition had significantly changed. In CTL culture, the cell viability was 95.2% with a total number of cells ranging from 686.7 to 31,281.7 × 10^6^. Among them, the number of CTLs was from 286.9 to 17,267.0 × 10^6^, accounting for 60.5%, increased 70.9- to 1513.3-fold. Meanwhile, in NK cell culture, the cell viability was 95.2%. The total number of cells reached from 661.8 to 10,105.5 × 10^6^, with a number of NK cells from 545.6 to 9062.9 × 10^6^, accounting for 86% of the cell population. The expansion of NK cells was impressively high with a 542.5-fold increase compared to the number of NK cells at seeding. Especially in NK cell culture, one sample exhibited the fold increase of 2374.8 times (Table 2). All cultures were negative for mycoplasma and bacteria/fungi. The endotoxin level was <0.5 EU/mL. 

### 2.3. The Relative Relationship of Immune Cell Expansion Ability to the Patient’s Age and Gender

In peripheral blood, there was a significant difference in the percentage of lymphocytes by gender, with 34.8% for female and 22.3% for male (*p*-value = 0.0013), by age, with 33.6% for patients below and 19.8% above 66 years old (*p*-value = 0.0002), by cancer type, with the highest in lung cancer (33.4%), then colon cancer (21.3%), and then liver cancer (18.7%) (*p*-value = 0.0006). However, there was no significant difference in the percentage of monocytes of the patients by gender, age, or type of cancer. The number of mononuclear cells was significantly lower in males compared to female patients (45 × 10^6^ vs. 67 × 10^6^, *p*-value = 0.0282). Moreover, the percentage of NK cells and CTLs was lower in males compared to females (*p*-value < 0.05). Meanwhile, there was no such significant difference between age groups.

After 20–21 days of culture, the total number of viable cells in the CTL culture differed significantly by gender, with a higher number of viable cells in female (10.1 ± 7.3 × 10^9^ cells) than compared to males (3.4 ± 2.8 × 10^9^ cells) (*p*-value = 0.0017). The percentage of CTLs among cell population accounted for 52.6% (corresponding with 2.0 ± 2.1 × 10^9^ cells) in males and 71.2% in females (corresponding to 7.0 ± 4.5 × 10^9^ cells) (*p*-value = 0.0019). The number of viable cells was three times higher (5.9 ± 4.5 × 10^9^ cells) in patients ≤66 years old (8.8 ± 6.8 × 10^9^) compared to the older patients (2.9 ± 2.7 × 10^9^ cells) (*p*-value = 0.0062). In the NK cell culture, the percentage of NK cells in the age above 66 years old was significantly higher than the age below 66 (90.6% vs. 82.1%) (*p*-value = 0.027). Similarly, the percentage of living cells was also higher in patients >66 years old compared to the younger patients (96.2% vs. 94.4%) (*p*-value = 0.0485).

The expansion ability of immune cells was very different between female and male patients. The number of total immune cells in CTL culture increased 87.8-fold in males, two times less than in females, with 171-fold (*p*-value = 0.0202). There was no significant difference in the number of CTLs or NK cells by gender. Meanwhile, in NK cell culture, the results for fold changes were opposite with the number of medium-specific NK cells increasing in males; 723-fold in males compared to 283-fold in females (*p*-value = 0.0298). There was no difference in the total number of immune cells. In CTL culture, the fold increases of total cell numbers and medium-specific cell numbers were 2.3 times higher in the patients <66 years old compared to the age group >66 years (*p*-value < 0.008).

### 2.4. Safety of Ex Vivo Expanded Immune Cell Transfusion

The results illustrated that immunotherapy is safe. There were no adverse events in the process off collecting peripheral blood and no severe complications (vomiting, fever, allergies) occurred before or after autologous immune cells transplantation.

The visual analog fatigue scale (VAFS) values decreased significantly from a mean of 8.0 ± 0.8 at baseline to 4.7 ± 1.1 at six months after intervention and 3.6 ± 0.8 at 12 months after intervention (*p*-value < 0.05). The Eastern Cooperative Oncology Group (ECOG) scales experienced a downward trend during the follow-up period (Table 1), which dropped from 3.2 points at baseline to 2.0 points at six months, and 1.9 points at 12 months (Table 1 and Table 3).

Changes in quality of life (QoL) were assessed at six months and 12 months after immune cells transplantation. The global health status improved during the follow-up (60.0 vs 29.2, *p*-value < 0.05). There was a significant difference for the measurement at six and 12 months after infusion of immune cells compared to baseline value (*p*-value < 0.05). Significant improvements were observed for the dimension of symptoms on fatigue, vomiting, pain, dyspnea, constipation, and diarrhea scales (*p*-value < 0.05). Regarding insomnia, there was a tendency for improvement (45.0 vs. 37.5, *p*-value >0.05). QoL had no change in the appetite loss after six months (57.5 vs. 42.5, *p*-value > 0.05) but improved after 12 months (57.5 vs. 27.5, *p*-value < 0.05). However, financial conditions deteriorated (62.5 vs 50.0, *p*-value < 0.05) (Table 4).

**Figure 1:**
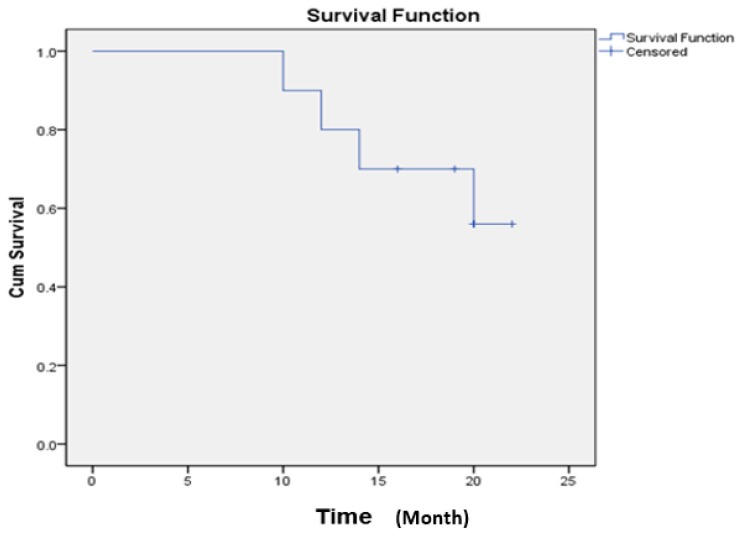
Survival time of patients enrolled in this study.

The mean overall survival (OS) rate at the last evaluation was 18.7 months, the 1-year overall survival (OS) rate was 80%, and the 2-year OS rate was 56% (Figure 1).

## 3. Discussion

Autologous immune cells transplantation has been widely used in the last two decades for cancer treatment. This method was proven to be safe and contributes to an increase of 30% in efficacy for cancer treatment when combined with other, more traditional, methods, such as surgery, chemotherapy, or radio therapy [27]. The crucial aspect for a successful application of this method is an adequate number of immune cells expanded from a very small number of relevant cells in the peripheral blood of cancer patients [28]. In most cases published, the doses of cells varied depended on the expansion method or the condition of the patient. Kananathan et al. [29] reported a rare case of advanced epithelioid sarcoma used in 7 autologous immune cell infusions containing an average number of NK cells and CTLs per each dose of 1880 × 10^6^ and 1760 × 10^6^, respectively, which constituted a more than 100-fold expansion compared with the initial number of cells at seeding. This patient had an overall survival rate of 25 months without any further chemotherapy [29]. In another case of a locally advanced carcinoma of the cervix, the average numbers of expanded NK cells and CTLs were 282.5 × 10^6^ and 478.5 × 10^6^, respectively. After six transfusions, a complete resolution of retroperitoneal lymph node was achieved with no evidence of the local lesion of the cervix [30]. A case report of a stage IV colonic cancer patient described an average fold expansion of NK cells and CTLs at 44- and 168-fold, respectively [31]. Meanwhile, our study achieved 962.5- and 730.6-fold change increases for NK cells and CTLs, respectively. A case report of a 15-year-old girl with Philadelphia chromosome positive acute lymphoblastic leukemia indicated that autologous immune cell infusion was efficient in erasing cancer cells when using it in combination with chemotherapy, and the achieved remission continued for five years after the end of the treatment. The average number of NK cell expansions in this case was 562 × 10^6^ [32].

Based on the method we had already developed [26], the immune cell expansion in this cohort study was carried out for three types of cancer (lung, liver, and colon), with a total of 29 sitting across patients. Compared to the literature, our results demonstrated that we were successful in expanding NK cells and CTLs with high average numbers of 2570.1 × 10^6^ and 4105.9 × 10^6^, respectively. The expansion of immune cells from liver cancer patients was lower compared to that of lung cancer patients. For colon cancer, there was only one patient rendering our results not suitable for further comparison. The percentage of selective cell populations in each expanded culture were rather high compared to the study previously reported. Our results also indicate that the immune cell expansion ability had a correlation with gender and age of the patients (*p* < 0.05), while the initial number of cultured cells or type of cancer did not seem to have a significant impact on their ability to be expanded.

All patients included in this study had stage IV malignancy. According to the European Organization for Research and Treatment of Cancer Quality of Life Questionnaire Version 3.0 (EORTC QLQ-C30), our study showed that the global health status of patients’ has improved after one year of monitoring. All scales of functional dimensions (physical, role, emotional, social, and cognitive) of the EORTC QLQ-C30 improved over time, resulting in high scores at the conclusion of the follow up period. This could be explained by the fact that the immune cell infusion has a significant positive effect on the change in social and family life aspects of our patients. Our results are consistent with those of authors who reported a significant improvement in the quality of life of these different scales during follow-up [24,28,29,30,31,32,35]. As for the symptom scales of EORTC QLQ-C30, they revealed a significant decrease in the severity of symptoms for fatigue, vomiting, pain, dyspnea, constipation, and diarrhea scales. There was a tendency to decrease in symptom severity for insomnia scales.

Based on some studies of lung cancer patients in Vietnam, fatigue is the most prevalent symptom, and about 30% of lung cancer patients rate this symptom from severe to very severe. Sustained fatigue was reported to negatively affect the quality of life and constituted an impediment to the functional status of the patients and their daily activities [36]. In our study, the fatigue mean value decreased by 42.7% after six months and by 54.5% 12 months after treatment. This demonstrates that AIET helped to improve the life in lung cancer patients. Liver cancer contributed significantly to the healthcare burden in Vietnam with an increasing volume of cases over the years. Hepatocellular carcinoma is a serious public health issue in Vietnam, with reported cases varying with age; however, adjusted incidence rates are generally thought to be greater than 20 per 100,000 people, compared to the prevalence reported for industrialized countries, which are estimated to be less than five per 100,000 people [37]. According to a report by Song Huy et al. [34], examining 24,091 Vietnamese patients with hepatocellular carcinoma (HCC), a large proportion of individuals (40.8% of the total) suffer from advanced HCC, only amenable to mere palliative care. Liver cancer patients enrolled in our study exhibited a poor state of health with an ECOG score of 3.2; however, after 12 months of treatment, these patients benefitted from an improved state of health and regained a significant degree of quality of life and independence.

Previous studies using autologous NK cells and CTLs for cancer treatment utilized highly variable doses of cells for infusion [22,23,24,25,27,28,29,30,31,32]. To date, there is no generally accepted or suggested limitation for the dose of immune cell transfusion [27,28]. Therefore, we opted in this study to collect all expanded cells and infuse them into the cancer patients. All patients included in this study reported no serious side effects of AIET. Even with the highest dose used in this study (3.12 × 10^10^ immune cells) in lung cancer patient (PT) 3, this patient benefitted from improved quality life with an ECOG score of 1 at the last evaluation. More importantly, the mean survival time for patients in our study was 18.7 months at 12 months after the transplantation, hence it increased more than twice compared to the estimated survival time at the point of enrollment. From a published report in early 2018 by Hoang et al. [26], the mean survival time of seven lung cancer patients were assessed at 17.1 ± 2 months and increased to 19.4 ± 1.7 months after treatment. The 1-year survival rate of our study was 80%, which was higher compared to other reports previously published [36,37]. Among all patients included in our study, the longest survival time to date was 36 months, achieved for the oldest female patient in our cohort at 87 years of age. At present, six out of 10 patients are still alive (Table 1).

## 4. Methods

### 4.1. Patients

#### 4.1.1. Inclusion Criteria

- Patients with confirmed diagnosis of colon, liver, or lung cancer by physician’s judgement.
- Eastern Cooperative Oncology Group performance status (ECOG/PS) ≥3.
- Patients signed the written informed consent form, which was approved by the Ethics Committee of Vinmec International Hospital in 18th November 2015, project identification code Vingroup JSC ĐT-00.

#### 4.1.2. Exclusion Criteria

- Severe health conditions, such as serious infection, autoimmune diseases, or the use of anti-rejection drugs, or T-cell lymphomas.

### 4.2. Study Design

An open-label, uncontrolled clinical trial. 

### 4.3. Research Setting and Duration

- The study was carried out at the Oncology Department, Vinmec Times City International Hospital, from March 2016 to June 2017.
- This study was conducted with the permission of the Vietnam Ministry of Health (Hanoi, Vietnam) (document no. 2517/BYT-KCB).

### 4.4. Cohort Size

- During the study period, 10 patients met the inclusion criteria.
- A total of 10 peripheral blood samples were marked as PT1 to PT10, corresponding to 10 patients (PT), 7 lung cancer patients were labeled as PT1 to PT7, 2 liver cancer patients were labeled as PT8 and PT9, and 1 colon cancer patient labeled as PT10.

### 4.5. Isolation and Large-Scale Expansion of NK Cells and CTLs From Peripheral Blood

The method for immune cell expansion had already been published [26]. In summary, peripheral blood mononuclear cells (PBMNCs) were obtained by density gradient centrifugation. using Ficoll-Paque (GE Healthcare, Sweden), and were cultured using BINKIT^®^ (Biotherapy Institute of Japan, Japan) at a density of 1 × 10^6^ cells/mL in the cell initial medium, supplemented with 5% of heat-inactivated autologous plasma, cultured in an anti-cluster of differentiation 16 (CD16) monoclonal antibody-immobilized culture flask for NK cell expansion and in anti-CD3 monoclonal antibody-immobilized flask for CTL expansion. After 3 days, the culture medium was changed and sub-cultured every 2–3 days in the subculture medium, supplemented with 5% of heat-inactivated autologous plasma, to maintain the concentration of 0.8–1.0 × 10^6^ cells/mL without discarding the old medium. When the number of cells increased logarithmically, the cultured cells were transferred into culture bags (Nipro, Japan) until the end of the culture. The cell processing center was set up in compliance with Good Manufacturing Practice (GMP) standards. The cells had to be administered within 14 h from cell processing, and were transported from the facility to the place where the patients were admitted for the infusion to assure optimum viability.

The phenotype of expanded cells and PBMNCs at baseline (day 0) and the end of culture were analyzed by flow cytometry. Monoclonal antibodies specific for CD3, CD8, CD56, and CD4 were conjugated with Pacific Blue, fluorescein isothiocyanate (FITC), R Phycoerythrin (PE), and Allophycocyanin-Alexa Flour 750 (APC-alexa Flour 750), respectively (Beckman Coulter, CA, USA), and the corresponding isotypes were used for the characterization of cell populations. Cells were analyzed by the Navios Cytometer (Beckman Coulter, USA), and data was acquired by Navios software, version 3.2, according to the manufacturer’s instructions.

### 4.6. Dosage and Duration

The total of expanded immune cells in one sitting were used to transfuse to the patients, so the dose was determined case by case.

One sitting of treatment consisted of one NK cell and one CTL infusion. The number of sittings was determined case by case, depending on the severity/staging/spread/organ type of cancer and general condition of patients. For one sitting, 50 mL of peripheral blood were collected. Immune cell treatments were given alone or in combination with the other conventional treatments, such as chemotherapy or radiotherapy, for optimal efficacy. For patients undergoing chemotherapy, this therapy needed to stop three days prior to the immune cell infusion and resumed three days after the infusion. The peripheral blood had to be collected before the start of radiotherapy. The cell infusions were planned to be applied 1 h before and after radiotherapy.

The expanded immune cells were transfused into the patients as an intravenous drip. About 70 mL of immune cell solution was transfused in within 15–60 min. On the day of infusion, the patients were advised to stay for 8 h in the hospital for observation.

### 4.7. Clinical Assessment

The visual analog fatigue scale (VAFS) was applied, which has been designed to describe the severity of fatigue experienced by patients with cancer [38]. The Eastern Cooperative Oncology Group (ECOG) illustrates a patient’s level of functioning in terms of their ability to care for themselves, perform daily activities, and their physical ability (walking, working, etc.) [39]. Quality of life was measured using EORTC QLQ-C30, which includes 30 items describing functional dimensions and dimension symptoms. The functional dimensions are composed of physical scales and emotional, cognitive, social, and professional activity. The symptom dimension consists of fatigue s, pain, and nausea/vomiting scales. In addition, we it has a global health scale; five scales describing simple symptoms (dyspnea, insomnia, loss of appetite, constipation, and diarrhea) and a scale assessing the perceived financial impact of the disease. According to the guidelines of the EORTC, scores on the items were converted to a scale of 0 to 100. A high score for a functional scale represents a healthy level of functioning and a high score for the overall health status represents a high quality of life, but a high score on a scale of the symptoms post represents a high level of symptomatology [40].

### 4.8. Statistical Analysis

Statistical analysis initially consisted of a description of our study population. Categorical variables were expressed in proportion, while quantitative variables were described by the mean value and their standard deviations. A paired t-test was used to assess the relationship between immune cell expansion ability and the patient’s age and gender. For the assessment of the quality of life, the student’s t-test for the comparison of the mean value paired data was used to identify a possible existence of differences in quality of life between the different parameters at the baseline, 6 months and 12 months after using AIET for the EORTC-C30 scale. The survival curves were illustrated using the Kaplan-Meier model. Data were analyzed using STATA Version 12.0 software.

## 5. Conclusions

In conclusion, we were successful in expanding the immune cells from the peripheral blood of colon, liver, and lung cancer patients. The number and quality of expanded cells satisfied the requirement for clinical use. The clinical trial demonstrated that this therapy is safe and feasible and can improve the quality of life for patients.

## Figures and Tables

**Table 1:** Clinicopathological data of the lung cancer patients enrolled in the study.

Patients	PT1	PT2	PT3	PT4	PT5	PT6	PT7	PT8	PT9	PT10
**Age**	84	64	30	53	66	62	40	69	80	59
**Gender**	F	F	F	F	M	M	F	M	M	F
**Cancer type**	Lung	Lung	Lung	Lung	Lung	Lung	Lung	Liver	Liver	Colon
**Histological Type**	AC	AC	AC	AC	SqC	AC	AC	HCC	HCC	AC
**Stage**	IV	IV	IV	IV	IV	IV	IV	IV	IV	IV
**Disease Stage (Metastatic or recurrent)**	M1m (bone, lung)	M0	M1m (lung, bone)	M1m (lung, brain, bone)	M1m (liver)	M1m (brain, bone)	M1m (bone)	M0	M1m (lung)	M1m (liver)
**Prior-treatment**	Chemo, targeted Taxol	Chemo Radio therapy	Targeted Taxol	Chemo, Targeted Taxol, Check point inhibitor	Chemo	Chemo Radio therapy chemo	Targeted Taxol	None	None	Chemo
**ECOG/PS at blood collection**	4	3	3	3	3	3	3	3	4	3
**ECOG/PS 6 months**	2	2	3	1	2	3	0	2	3	2
**ECOG/PS 12 months**	2	1	1	2	2	2	3	2	2	2
**Estimated survival prior to treatment (months)**	6	6	6	6	6	6	6	6	12	12
**Survival (time) at last evaluation**	+21	+19	+20	+19	−22	+18	−17	−16	−20	+16

Adenocarcinoma (AC); squamous cell carcinoma (SqC); hepatocellular carcinoma (HCC) + survival, – mortality; Eastern Cooperative Oncology Group (ECOG) performance status (ECOG/PS).

**Table 2:** Analysis of immune cells expansion, number of cell infusions, and cell dose.

	NK Cell Culture				CTL Culture		
Patients	Sitting Number	Total Cell Infused (×10^6^)/Sitting (Mean ± SD)	NK Cell Number (×10^6^)/Sitting (Mean ± SD)	Fold Increase of NK Cell (Mean ± SD)	Total Cell Infused (×10^6^)/Sitting (Mean ± SD)	CTL Number (×10^6^)/Sitting (Mean ± SD)	Fold Increase of CTL (Mean ± SD)
**PT1**	2	5238.3 ± 6472.2	4804.3 ± 6022.7	263.6 ± 351.1	3431.5 ± 1120.8	2359.6 ± 618.4	169.09 ± 30.9
**PT2**	2	2540.8 ± 1395.3	1311.5 ± 597.0	16.3 ± 57.5	21,106.2 ± 14,390.3	11,580.5 ± 8042.0	1014.9 ± 704.8
**PT3**	2	3637.2 ± 490.7	2943.8 ± 208.9	251.9 ± 17.9	12,178.3 ± 7436.6	10,007.9 ± 6532.9	863.5 ± 563.7
**PT4**	4	1787.6 ± 329.6	1553.8 ± 315.9	169.1 ± 35.7	9550.9 ± 1667.1	6389.2 ± 1502.8	492.7 ± 85.5
**PT5**	4	5935.5 ± 3418.2	4244.9 ± 2487.7	892.3 ± 1003.9	4862.9 ± 3251.5	2392.2 ± 1991.2	602.6 ± 346.4
**PT6**	2	3673.9 ± 461.0	3213.0 ± 506.4	1451.3 ± 228.7	4048.1 ± 723.3	2545.6 ± 394.9	454.3 ± 70.5
**PT7**	1	3697.3	3077.9	524.3	7241.4	5288.6	595.97
**PT8**	2	3405.8 ± 1367.8	2816.2 ± 1200.6	231.5 ± 143.9	6819.0 ± 5094.1	5581.5 ± 4283.0	284.19 ± 156.2
**PT9**	9	1870.6 ± 621.9	1658.3 ± 566.7	623.4 ± 401.3	2377.5 ± 1913.8	1059.5 ± 900.8	360.27 ± 333.1
**PT10**	1	4401.7	3555.5	962.49	8334.7	6015.4	730.57
Mean value *		3248.2	2616.9	548.9	6693.5	4251.9	505.8
Min value^*^		661.8	545.6	15.3	686.7	286.9	70.9
Max value^*^		10,105.5	9062.9	2374.9	31,281.7	17,267.04	1513.3

* Mean, min, and max values were calculated from a total of 29 sittings.

**Table 3:** Visual analog fatigue scale and Eastern Cooperative Oncology Group values after autologous immune enhancement therapy (AIET).

Items	Means ± Standard Deviation		
	Baseline	6 months	12 months
Visual analog fatigue scale	8.0 ± 0.8	4.7 ± 1.1 *	3.6 ± 0.8 *
Eastern Cooperative Oncology Group	3.2 ± 0.4	2.0 ± 0.7 *	1.9 ± 0.9 *

* *p*-value < 0.05.

**Table 4:** Quality of life improvement after immune cells transplantation.

Items	Means ± Standard Deviation		
	Baseline	6months	12 months
Global Health Status	29.2 ± 9.8	59.2 ± 8.3 *	60 ± 6.6 *
Physical functioning	30.6 ± 7.8	50.7 ± 15.1 *	62.0 ± 16.9 *
Role functioning	25.0 ± 11.8	50.0 ± 13.6 *	58.3 ± 8.8 *
Emotional Functioning	34.2 ± 9.2	61.7 ± 9.0 *	76.7 ± 17.0 *
Cognitive functioning	31.7 ± 14.6	55.0 ± 13.7 *	61.7 ± 11.2 *
Social functioning	26.7 ± 11.7	51.7 ± 12.3 *	56.7 ± 14.1 *
**Symptom scales**			
Fatigue	56.7 ± 9.5	32.5 ± 8.3 *	25.8 ± 7.3 *
Nausea and Vomiting	26.3 ± 9.2	13.8 ± 10.9 *	7.5 ± 6.5 *
Pains	43.8 ± 15.9	30.0 ± 8.7 *	27.5 ± 11.5 *
Dyspnea	40.0 ± 21.1	22.5 ± 14.2 *	20.0 ± 15.8 *
Insomnia	45.0 ± 10.5	35.0 ± 12.9	37.5 ± 13.2
Appetite loss	57.5 ± 16.9	42.5 ± 20.6	27.5 ± 14.2 *
Constipation	27.5 ± 14.2	10.0 ± 12.9 *	5.0 ± 10.5 *
Diarrhea	20.0 ± 10.5	2.5 ± 7.9 *	0
Financial difficulties	62.5 ± 13.2	47.5 ± 7.9 *	50.0 *

* *p*-value < 0.05.

## Abbreviations

AC	Adenocarcinoma
AIET	Autologous immune enhancement therapy
BIJ	Biotherapy Institute of Japan
BINKIT	Immune cell expansion kit of Biotherapy Institute of Japan
CD	Cluster of differentiation
CIK cells	Cytokine-induced killer cells
CTL	Cytotoxic T Lymphocyte
D	Day of culture
DC	Dendritic cell
ECOG/PS scale	Eastern Cooperative Oncology Group/Performance Status scale
EORTC QLQ-C30	Organization for Research and Treatment of Cancer Quality of Life Questionnaire Version 3.0
HCC	Hepatocellular carcinoma
IL	Interleukin
MHC	Major histocompatibility complex
NK cell	Natural killer cell
OS	Overall survival
PBMNC	Peripheral blood mononuclear cell
PT	Patient
QoL	Quality of life
SqC	Squamous cell carcinoma
TCR	T-cell receptors
VAFS	Visual analog fatigue scale
